# SCMCRYS: Predicting Protein Crystallization Using an Ensemble Scoring Card Method with Estimating Propensity Scores of P-Collocated Amino Acid Pairs

**DOI:** 10.1371/journal.pone.0072368

**Published:** 2013-09-03

**Authors:** Phasit Charoenkwan, Watshara Shoombuatong, Hua-Chin Lee, Jeerayut Chaijaruwanich, Hui-Ling Huang, Shinn-Ying Ho

**Affiliations:** 1 Institute of Bioinformatics and Systems Biology, National Chiao Tung University, Hsinchu, Taiwan; 2 Department of Computer Science, Bioinformatics Research Laboratory, Chiang Mai University, Chiang Mai, Thailand; 3 Department of Biological Science and Technology, National Chiao Tung University, Hsinchu, Taiwan; University of Akron, United States of America

## Abstract

Existing methods for predicting protein crystallization obtain high accuracy using various types of complemented features and complex ensemble classifiers, such as support vector machine (SVM) and Random Forest classifiers. It is desirable to develop a simple and easily interpretable prediction method with informative sequence features to provide insights into protein crystallization. This study proposes an ensemble method, SCMCRYS, to predict protein crystallization, for which each classifier is built by using a scoring card method (SCM) with estimating propensity scores of *p*-collocated amino acid (AA) pairs (*p* = 0 for a dipeptide). The SCM classifier determines the crystallization of a sequence according to a weighted-sum score. The weights are the composition of the *p*-collocated AA pairs, and the propensity scores of these AA pairs are estimated using a statistic with optimization approach. SCMCRYS predicts the crystallization using a simple voting method from a number of SCM classifiers. The experimental results show that the single SCM classifier utilizing dipeptide composition with accuracy of 73.90% is comparable to the best previously-developed SVM-based classifier, SVM_POLY (74.6%), and our proposed SVM-based classifier utilizing the same dipeptide composition (77.55%). The SCMCRYS method with accuracy of 76.1% is comparable to the state-of-the-art ensemble methods PPCpred (76.8%) and RFCRYS (80.0%), which used the SVM and Random Forest classifiers, respectively. This study also investigates mutagenesis analysis based on SCM and the result reveals the hypothesis that the mutagenesis of surface residues Ala and Cys has large and small probabilities of enhancing protein crystallizability considering the estimated scores of crystallizability and solubility, melting point, molecular weight and conformational entropy of amino acids in a generalized condition. The propensity scores of amino acids and dipeptides for estimating the protein crystallizability can aid biologists in designing mutation of surface residues to enhance protein crystallizability. The source code of SCMCRYS is available at http://iclab.life.nctu.edu.tw/SCMCRYS/.

## Introduction

Knowledge of three-dimensional protein structures is crucial when investigating protein functions. The structural knowledge is considered to be important when designing drugs involving the protein functions [1]. In general, X-ray crystallography and nuclear magnetic resonance spectroscopy are commonly used for determining the structures of proteins. Approximately 80% of the protein structures in Protein Data Bank (PDB) were obtained by using the X-ray crystallography method [2]. In fact, these two approaches involve very complex, time-consuming, laborious and expensive processes. Because of the difficulties in determining the crystal structures, the current protocol yields only a 30% success rate [3]. Thus, many researchers take advantage of computational approaches to directly predicting protein crystallization.

Canaves et al. [4] and Goh et al. [5] have proposed methods for extracting informative features to predict protein crystallization. Many sequence-based computational methods, including OB-Score [6], SECRET [7], CRYSTALP [8], XtalPred [9], ParCrys [10], CRYSTALP2 [11], SVMCRYS [12], PPCpred [13] and RFCRYS [14], predict protein crystallization, as shown in Table 1. Both support vector machine (SVM) [7], [12], [13] and the ensemble mechanism [13], [14] are well-known techniques to enhance prediction accuracy. Because of the different design aims and benchmarks used, it is not easy to assess which method and features are the most effective. From the study in [14] and Table 1, we can see that the SVM_POLY method (see the work [13]) using SVM has the highest accuracy among the non-ensemble methods. This method is one of the four SVM predictors that are integrated into PPCpred [13]. The state-of-the-art ensemble methods PPCpred and RFCRYS have high prediction accuracies using the SVM and Random Forest classifiers, respectively. PPCpred utilizes a comprehensive set of inputs that are based on energy and hydrophobicity indices, the composition of certain amino acid types, predicted disorder, secondary structure, solvent accessibility, and the content of certain buried and exposed residues [13]. RFCRYS predicts the protein crystallization by utilizing the mono-, di- and tri-peptide compositions; the frequencies of amino acids in different physicochemical groups; the isoelectric point; the molecular weight; and the length of the protein sequences [14]. However, the mechanism of these two ensemble classifiers suffers from low interpretability for biologists. It is not clear which sequence features provide the essential contribution to the high prediction accuracy.

**Table 1:** Some existing methods for predicting protein crystallization from sequences.

Method	Classifier	Sequence Features (no. of feature types)	Single/Ensemble	Year
OB-Score [6]	Single Threshold	PCP (1)	Single	2006
SECERT [7]	SVM	AAC, DPC, TPC (3)	Single	2006
CRYSTALP [8]	Naïve Bayes	AAC, PAAC (2)	Single	2007
XtalPred [9]	Logarithm Method	AAC, PCP, SS (3)	Single	2007
ParCrys [10]	Parzen Window Density Estimator	AAC, PCP, Low complexity region (3)	Single	2008
CRYSTALP2 [11]	Gaussian radial basis function network	AAC, DPC, TPC, PAAC, PCP (5)	Single	2009
SVMCRYS [12]	SVM	AAC, TPC, PCP, SS (4)	Single	2010
PPCpred [13]	SVM	PCP, AAC, SS, Disorder, Solvent accessibility (5)	Ensemble	2011
RFCRYS [14]	Random Forest	AAC, DPC, TPC, PCP, Sequence Length (5)	Ensemble	2012
SCMCRYS	SCM	PAAC (1)	Ensemble	This study

SS is defined as secondary structure.

AAC is defined as amino acid composition.

DPC is defined as dipeptide composition.

TPC is defined as tripeptide composition.

PCP is defined as physicochemical properties.

PAAC is defined as *p*-collocated amino acid pair composition.

PseAAC is defined as Pseudo amino acid composition.

Rather than increasing both the complexity of prediction methods and the number of feature types while pursuing high accuracy, the motivation of this study is to provide a simple and highly interpretable method with a comparable accuracy from the viewpoint of biologists. The *p*-collocated AA pairs (*p* = 0 for a dipeptide) are shown to be significant in influencing or enhancing protein crystallization because of the impact of folding corresponding to the interaction between local AA pairs [8], [11]. The *p*-collocated AA pairs provide the additional information on which the interaction between local AA pairs reflects besides the simple AA composition. This study proposes an ensemble method, SCMCRYS, to predict protein crystallization in which each classifier is built by using a scoring card method (SCM) [15] with estimating propensity scores of *p*-collocated AA pairs to be crystallizable. Compared to SCM using dipeptide composition in [15], the ensemble classifier of SCMCRYS makes the best use of p-collocated AA pairs. The rules for deciding whether a protein is crystallizable in the SCM classifier and SCMCRYS are very simple according to a weighted-sum score and a voting method from a number of SCM classifiers, respectively. However, the experimental results show that the SCM classifier is comparable to SVM_POLY and the SVM-based classifiers with *p*-collocated AA pairs. The SCMCRYS method is comparable to the state-of-the-art ensemble methods PPCpred and RFCRYS.

The propensity scores of dipeptides and amino acids to be crystallizable are highly correlated with the crystallization ability of sequences and can provide insights into protein crystallization. Furthermore, the propensity scores of amino acids can also reveal the relationship between crystallizability and physicochemical properties such as solubility, molecular weight, melting point and conformational entropy of amino acids. This study also proposes a mutagenesis analysis method for illustrating the additional advantage of SCM. We investigate the mutagenesis analysis for enhancing protein crystallizability based on the estimated crystallizability scores, solubility scores [15], and physicochemical properties of amino acids. The analysis result reveals the hypothesis that the mutagenesis of surface residues Ala and Cys has large and small probabilities of enhancing protein crystallizability in applying protein engineering approaches.

## Results and Discussion

In this study, the crystallizable and non-crystallizable proteins are predicted by the SCM-based ensemble method SCMCRYS. We utilize training and test datasets called CRYS-TRN and CRYS-TEST, respectively, derived from the work [13]. The SCM, SVM and SCMCRYS classifiers using the features of *p*-collocated AA pair information were constructed using CRYS-TRN for predicting each protein in CRYS-TEST. The prediction performances are evaluated in terms of the test accuracy, Mathew's correlation coefficient (MCC), Specificity and Sensitivity. In the experiments of evaluation and performance comparisons, we first establish the SCM classifiers to predict protein crystallization by utilizing the *p*-collocated AA pairs. Then, these SCM classifiers are further integrated into the proposed SCM-based ensemble method SCMCRYS. We also propose the SVM classifiers based on the same *p*-collocated AA pair composition for performance comparisons. To compare with existing prediction methods, the SCM and SCMCRYS are regarded as a single classifier and ensemble method, respectively. Finally, the propensity scores of *p*-collocated AA pairs to be crystallizable derived from the SCM classifier are utilized to investigate factors for enhancing the crystallization of proteins based on knowledge of protein engineering.

### Performance of SCM using p-collocated AA pairs

The SCM method consists of two stages. The first is the initiation stage using a statistical approach to obtaining the initial propensity scores of *p*-collocated AA pairs. The second is the optimization stage optimizing the initial propensity scores by utilizing an intelligent genetic algorithm [16]. The SCM method without using the optimization stage is named Init-SCM.

The prediction performances of Init-SCM using the *p*-collocated AA pairs where *p* varies from 0 to 9 are shown in Table 2. The mean performance of a single SCM classifier is the test accuracy of 70.97%, MCC = 0.28, Sensitivity = 0.29, and Specificity = 0.91. The best classifiers are the SCMs using relative small values of *p*, but the difference of accuracies is very small. For performance comparisons with existing prediction methods, the used benchmark dataset is not balanced that the number of positive samples (crystallizable) is smaller than that of negative samples (non-crystallizable). Therefore, the sensitivity accuracy is much lower than the specificity accuracy. The threshold value of determining the predicted class can be used to adjust the sensitivity and specificity accuracies if one prefers Sensitivity to Specificity.

**Table 2:** Performance of the Init-SCM method using the *p*-collocated AA pairs.

*p-*collocated	Test Accuracy (%)	MCC	Sensitivity	Specificity
*p* = 0	71.47	0.30	0.33	0.91
*p* = 1	71.72	0.30	0.32	0.92
*p* = 2	71.05	0.29	0.32	0.91
*p* = 3	71.42	0.30	0.37	0.89
*p* = 4	71.02	0.29	0.33	0.90
*p* = 5	71.14	0.28	0.27	0.93
*p* = 6	70.74	0.27	0.29	0.92
*p* = 7	70.21	0.25	0.19	0.96
*p* = 8	70.77	0.27	0.21	0.96
*p* = 9	70.13	0.26	0.31	0.90
Mean	70.97±0.52	0.28±0.02	0.29±0.05	0.91±0.02

**Figure 1:**
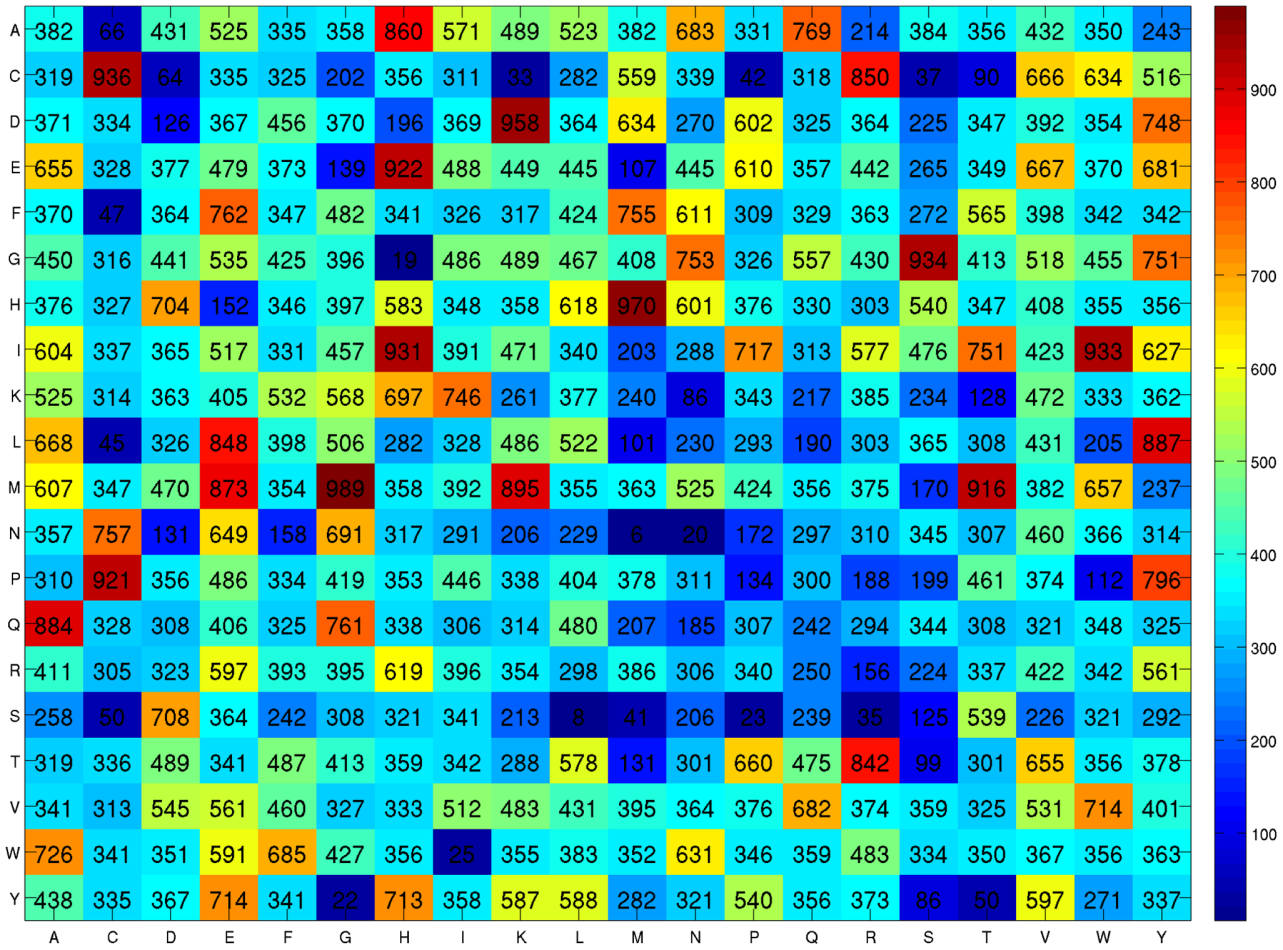
Heat map of the propensity scores of dipeptides obtained from the SCM method.

Due to the non-deterministic characteristic of genetic algorithms which use randomicity mechanism resulting in non-constant results, 10 independent runs were conducted to generate 10 SCM classifiers for each value of *p* where *p* = 0, 1, …, 9. The mean performances of SCM using the *p*-collocated AA pairs are shown in Table 3. The best SCM classifier is the one using dipeptide composition (*p* = 0) that the test performance is 73.90±0.57%, MCC  = 0.38±0.02, Sensitivity  = 0.45±0.03, and Specificity  = 0.88±0.01. The optimization stage improves SCM with dipeptide composition that the test accuracy increases from 71.47% to 73.90%, and the MCC value increases from 0.30 to 0.38. In the following analysis, the propensity scores of dipeptides obtained from the best result of SCM are adopted, as shown in Figure 1.

**Table 3:** Mean performance of the SCM method using the *p*-collocated AA pairs.

*p-*collocated	Test Accuracy (%)	MCC	Sensitivity	Specificity
*p* = 0	73.90±0.57	0.38±0.02	0.45±0.03	0.88±0.01
*p* = 1	72.63±0.77	0.35±0.02	0.46±0.03	0.86±0.02
*p* = 2	71.28±0.90	0.32±0.01	0.46±0.03	0.84±0.03
*p* = 3	73.30±0.59	0.37±0.01	0.49±0.03	0.86±0.02
*p* = 4	73.14±0.47	0.37±0.01	0.48±0.02	0.86±0.01
*p* = 5	71.10±0.48	0.32±0.01	0.47±0.02	0.83±0.02
*p* = 6	72.78±0.47	0.36±0.01	0.49±0.03	0.85±0.02
*p* = 7	71.73±0.59	0.33±0.01	0.47±0.03	0.84±0.02
*p* = 8	72.85±0.40	0.36±0.01	0.46±0.04	0.86±0.02
*p* = 9	72.55±0.84	0.36±0.01	0.49±0.03	0.85±0.03

**Figure 2:**
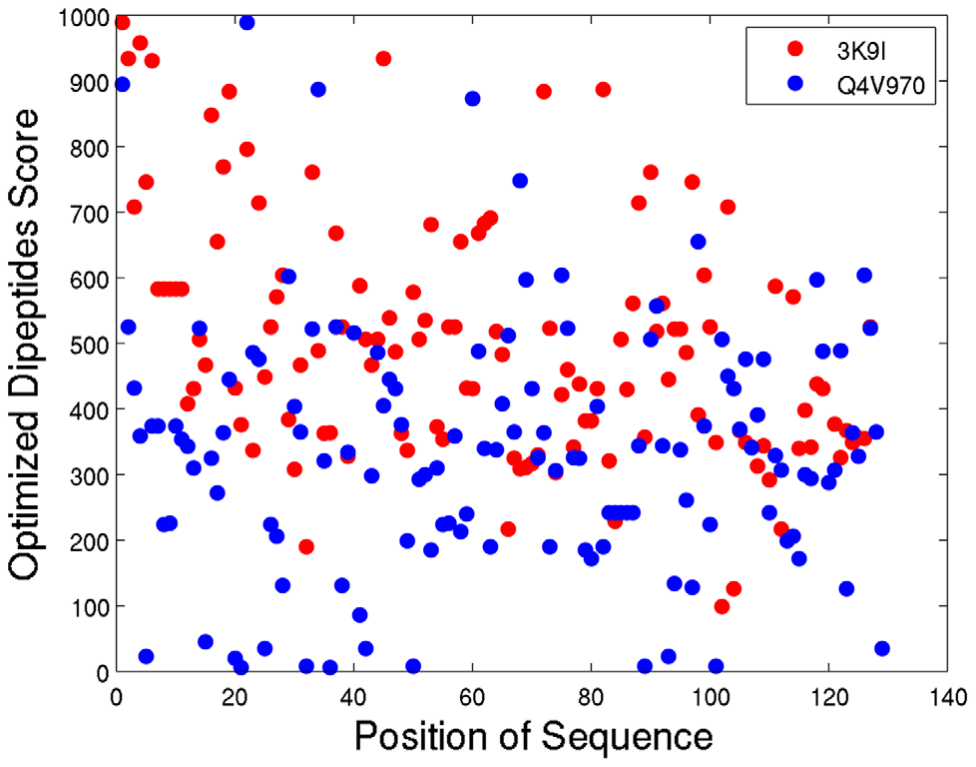
Distribution of locations of high-score dipeptides on the two typical sequences 3K9I and Q4V970. The distribution of locations of high-score dipeptides on the two typical sequences 3K9I and Q4V970 correctly predicted as crystallizable and non-crystallizable proteins, respectively.

To investigate the possibility that the top-ranked dipeptides with high crystallizability scores tend to cluster in a certain region, we conducted an experiment for investigating the distribution of locations of high-score dipeptides in protein sequences. Figure 2 shows the distribution of locations of high-score dipeptides on the two typical sequences 3K9I and Q4V970 correctly predicted as crystallizable and non-crystallizable proteins with sequence scores 505.93 and 336.60, respectively. The result shows that both high-score and low-score dipeptides were uniformly distributed on the sequences. Furthermore, the number of high-score dipeptides in the crystallizable protein is more than that of the non-crystallizable protein. From this result, it might be observed that top-ranked dipeptides do not tend to cluster in a certain region and crystallizability is a global property of sequences for general proteins.

### Performance comparisons between SCMCRYS and existing methods

To make the best use of *p*-collocated AA pairs, the proposed SCMCRYS method is designed to be an SCM-based ensemble classifier consisting of 100 SCM classifiers with *p* = 0 to 9 where each value of *p* corresponds to 10 SCM classifiers. SCMCRYS yields a test accuracy of 76.1%, MCC  = 0.44, Sensitivity  = 0.46, and Specificity  = 0.91. The ensemble approach of SCMCRYS improves the test accuracy from 73.9% to 76.1%, compared with SCM with dipeptide composition. The performance comparisons of SCMCRYS with existing prediction methods are shown in Table 4.

**Table 4:** Comparisons of the proposed method SCMCRYS with existing classifiers.

Classifiers	Type	Test Accuracy (%)	MCC	Sensiti vity	Specifi city
CRYSTALP2^a^	single	55.3	0.19	0.74	0.46
SVMCRYS^a^	single	56.3	0.21	0.75	0.47
SVM_POLY^a^	single	74.6	0.40	0.48	0.88
SVM_DPC	single	77.55	0.47	0.45	0.94
SCM (Dipeptide)	single	73.90	0.38	0.45	0.88
PPCpred^a^	ensemble	76.8	0.47	0.61	0.85
RFCRYS^a^	ensemble	80.0	0.53	0.51	0.95
SCMCRYS	ensemble	76.1	0.44	0.46	0.91

a Results come from the work RFCRYS [14].

The reported results of existing methods in Table 4 come directly from the work RFCRYS [14]. The compared non-ensemble prediction methods with SCM are CRYSTALP2 [11], SVMCRYS [12], SVM_POLY [13], and SVM with dipeptide composition (SVM_DPC) presented in this study. The best published method among these non-ensemble classifiers is the SVM_POLY method with an accuracy of 74.6%, which uses SVM with selected physiochemical properties, amino acids compositions, and secondary structure information. CRYSTALP2 utilizes the normalized Gaussian radial basis function network with the features of the *p*-collocated AA pairs and some physicochemical properties of amino acids. SVMCRYS utilizes SVM with the 116 features of amino acid composition, tripeptide composition, secondary structure, and physicochemical properties. The highest accuracy of 77.55% among these non-ensemble classifiers comes from SVM_DPC. The SCM method with 73.90% is slightly worse than SVM_POLY and SVM_DPC, and much better than CRYSTALP2 (55.3%) and SVMCRYS (56.3%).

The best ensemble method is RFCRYS with 80.0% using Random Forest with several types of complemented features: the mono-, di- and tri-peptide compositions, the frequencies of amino acids in different physicochemical groups, the isoelectric point, the molecular weight, and the length of protein sequences [14]. The second best method is PPCpred [13] with a test accuracy of 76.8% using a comprehensive set of features generated using several information sources. From these results, we can derive the following findings.

It can be well recognized that SVM_POLY obtains better results (74.6%) than SCM because the former classifier was developed using several types of complemented features (without using dipeptide composition) and SVM having a more complicated decision boundary. Notably, the SCM classifier (73.9%) uses a single type of features (i.e., dipeptide composition) and a single threshold value as a decision boundary. The ensemble classifier SCMCRYS (76.1%) is also comparable to the two ensemble classifiers PPCpread (76.8%) and RFCRYS (80.0%). These results reveal that the proposed SCM method with the propensity scores of dipeptides performs well considering the two objectives: maximizing interpretability of both classifier and used features.

### Comparison between SCM and SVM using p-collocated AA pairs

The SVM method is effective in predicting protein crystallization [7], [12], [13]. We examine the performance of SVM with a radial basis kernel function using the same *p*-collocated AA pair composition (PAAC) and the amino acid composition (AAC) for predicting protein crystallization. We used the LibSVM package [17] to perform all SVM experiments. The values of cost and gamma parameters of the SVM classifier are determined by using a grid search with 10-fold cross-validation (10-CV). From Table 5, the SVM+ACC classifier obtained a test accuracy of 73.12%, MCC = 0.35, Sensitivity = 0.38 and Specificity = 0.91. We also find that all of the SVM+PAAC classifiers outperform the SVM+AAC classifier. These results emphasize the superiority of PAAC over ACC in predicting protein crystallizability. The best classifier of SVM+PAAC is obtained by using *p* = 6, which yields a test accuracy of 77.69%, MCC = 0.47, Sensitivity = 0.50 and Specificity = 0.91. There is no existing method of using the classifier SVM+PACC in Table 4. The performance of this classifier SVM+PAAC is also better than previously reported non-ensemble classifiers such as SVM_POLY. Considering the case of our interest, i.e., *p* = 0, the SVM classifier using dipeptide composition achieves a good test accuracy of 77.55%, MCC = 0.47, Sensitivity = 0.45 and Specificity = 0.94.

**Table 5:** Performances of SVM using amino acid composition (AAC) and *p-*collocated AA pairs.

Feature Name	Test Accuracy (%)	MCC	Sensitivity	Specificity
AAC	73.12	0.35	0.38	0.91
*p* = 0	77.55	0.47	0.45	0.94
*p* = 1	77.02	0.46	0.49	0.91
*p* = 2	76.57	0.44	0.47	0.91
*p* = 3	76.65	0.44	0.44	0.93
*p* = 4	77.02	0.45	0.45	0.93
*p* = 5	76.46	0.44	0.48	0.91
*p* = 6	77.69	0.47	0.50	0.91
*p* = 7	76.37	0.44	0.44	0.93
*p* = 8	76.82	0.45	0.47	0.92
*p* = 9	77.52	0.47	0.51	0.91
Mean (*p* = 0,1, …, 9)	76.967	0.453	0.47	0.92

We propose the classifier SVM+PACC achieving the best accuracy in predicting protein crystallization using the benchmark dataset. It also reveals that the proposed SCM classifier using dipeptide (73.90%) is very promising, compared to the SVM classifier (77.55%) considering the simplicity, interpretability, and implementation. The SCM classifier is more suitable method for protein crystallization analysis because the biological meanings embedded in the propensity score of dipeptides and amino acids are the most desirable, discussed below.

### Propensity scores of amino acids

**Figure 3:**
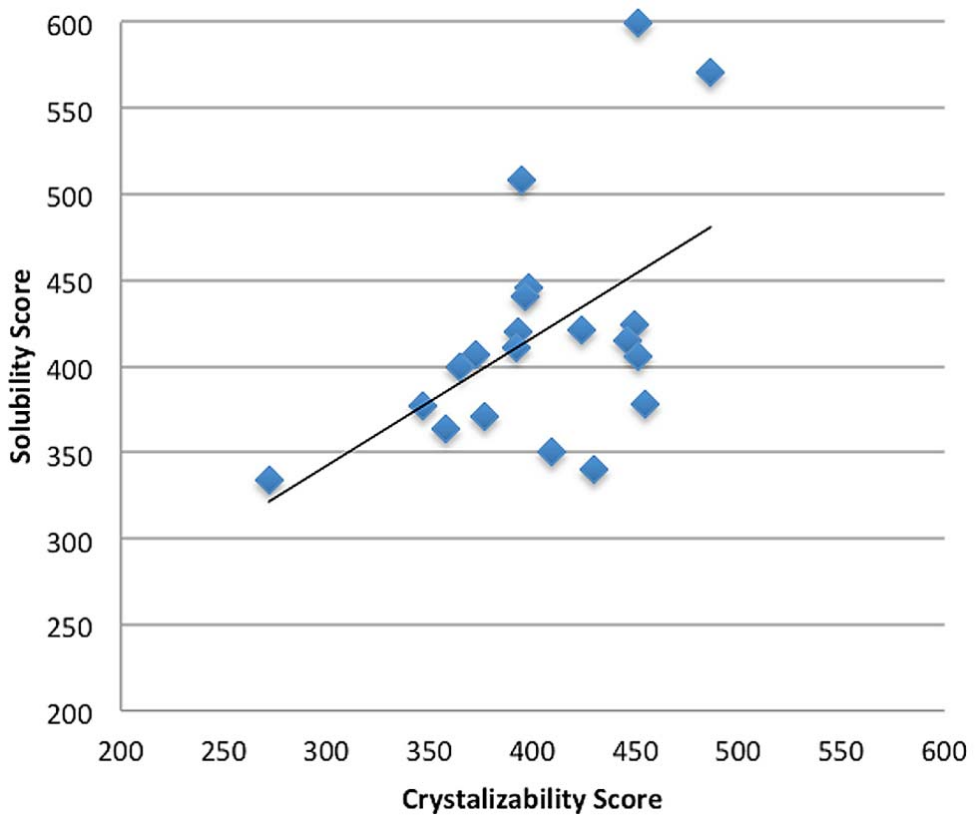
The scatter plot of correlation between solubility scores and crystallizability. **scores where R = 0.52.**

The 20 propensity scores of amino acids to be crystallizable derived from the scores of dipeptides (Figure 1) are shown in Table 6. Glu, Gly, Ala, His and Val are the five top-ranked amino acids to be crystallizable, and Ser, Asn, Cys, Gln and Pro are the five top-ranked amino acids to be non-crystallizable. Protein solubility is strongly correlated with the proteins' probability of yielding crystals [11], [15], [18]. The SCM method has been proposed to predict protein solubility [15]. The propensity scores of amino acids to be soluble derived from the optimized solubility scores of dipeptides [15] are also given in Table 6. Because the solubility is influenced by various condition factors such as temperature, pH, buffer concentration, and various additives, the solubility scores of amino acids are regarded as a set of generic propensities. Figure 3 shows the scatter plot of correlation between solubility and crystallizability scores of amino acids, and the Pearson's correlation R = 0.52. The ranks of propensity scores (crystallizability, solubility) for Glu, Gly and Ala are (1, 2), (2, 14) and (3, 1), respectively (Table 6). We can find that Glu and Ala are promising amino acids in applying the protein engineering approach to enhancing crystallizability considering both solubility and crystallizability scores. Similarly, Cys, Asn and Ser have high propensities to be non-crystallizable that the ranks of Cys, Asn and Ser are (18, 17), (19, 15) and (20, 20), respectively.

**Table 6:** The propensity scores of amino acids to be crystallizable and related physicochemical properties.

Amino acid	Crystallizability Score (rank)	Solubility Score (rank)	Melting point (rank)	Molecular weight (rank)	Conformational Entropy (rank)
E-Glu	486.38 (1)	570.85 (2)	249 (13)	147.13 (14)	1.81 (17)
G-Gly	454.90 (2)	378.05 (14)	290 (5)	75.07 (1)	0 (1)
A-Ala	451.38 (3)	599.42 (1)	297 (3)	89.09 (2)	0 (1)
H-His	451.23 (4)	406.18 (12)	277 (10)	155.16 (16)	0.96 (9)
V-Val	449.23 (5)	424.17 (6)	293 (4)	117.15 (5)	0.51 (4)
I-Ile	445.63 (6)	414.75 (9)	284 (7)	131.17 (12)	0.89 (8)
Y-Tyr	429.83 (7)	339.80 (19)	344 (1)	181.19 (19)	0.98 (10)
M-Met	423.63 (8)	420.88 (7)	283 (8)	149.21 (6)	1.61 (14)
W-Trp	408.88 (9)	350.00 (18)	282 (9)	204.23 (20)	0.98 (10)
K-Lys	398.30 (10)	445.27 (4)	224 (17)	146.19 (13)	1.94 (18)
L-Leu	395.95 (11)	440.73 (5)	337 (2)	131.17 (8)	0.78 (7)
D-Asp	394.53 (12)	507.95 (3)	270 (11)	133.10 (11)	1.25 (12)
F-Phe	392.83 (13)	420.12 (8)	284 (6)	165.19 (17)	0.58 (6)
T-Thr	392.45 (14)	411.02 (10)	253 (12)	119.12 (15)	1.63 (15)
R-Arg	376.90 (15)	370.58 (16)	238 (14)	174.20 (18)	2.03 (19)
P-Pro	372.28 (16)	406.23 (11)	222 (18)	115.13 (4)	0 (1)
Q-Gln	364.80 (17)	400.02 (13)	185 (19)	146.15 (9)	2.11 (20)
C-Cys	357.43 (18)	363.83 (17)	178 (20)	121.16 (7)	0.55 (5)
N-Asn	346.48 (19)	376.65 (15)	236 (15)	132.12 (10)	1.57 (13)
S-Ser	271.93 (20)	334.10 (20)	228 (16)	105.09 (3)	1.71 (16)
R	1.00	0.52	0.54	0.05	−0.32
R_1_	1.00	0.69	0.61	−0.12	−0.40
R_2_	1.00	0.93	0.90	0.30	−0.60

R is correlation between crystallizability scores and other physicochemical properties of amino acids.

R_1_ is correlation between crystallizability scores and other physicochemical properties of sequences in a training dataset.

R_2_ is correlation between crystallizability scores and other physicochemical properties of sequences belonging to the set consisting of 20 and 20 sequences with the highest and lowest crystallizability scores, respectively.

To further understand the relationship between crystallizability and solubility scores of protein sequences obtained from using SCM, let R_1_ be the Pearson's correlation between two sets of sequence scores. The sequence scores are the average of propensity scores or physicochemical properties of amino acids in a sequence. Additionally, let R_2_ be the Pearson's correlation between two sets of 40 sequence scores. The difference between R_1_ and R_2_ is that only 20 and 20 sequences of having the highest and lowest sequence scores with high confidence of estimation, respectively, are considered instead of the whole data set. We calculate the solubility and crystallizability scores of protein sequences in the training data set CRYS-TRN. The correlations between the solubility and crystallizability scores of sequences are R_1_ = 0.69 and R_2_ = 0.93. The high correlation agrees that protein solubility is strongly correlated with the proteins' probability of yielding crystals. The high correlation also reveals that SCM is reliable in producing the solubility and crystallizability scores.

### Relationship between crystallization scores and physicochemical properties

The crystallization behaviours are affected by the biochemical and biophysical properties of proteins such as conformational homogeneity, solubility and stability [18]. To further investigate the relationship between the crystallization scores and physicochemical properties of amino acids, we analysed all the 531 physicochemical properties in the AAindex database [15], [19]. The five top-ranked physiochemical properties having the largest absolute correlation values are given in Table 7. The property AURR980101 described as “Normalized positional residue frequency at helix termini N4′” is at rank 1 with Pearson's correlation R = 0.61. The property MAXF760106 described as “Normalized frequency of alpha region” is at rank 2 with correlation R = −0.57. These two properties are related to the residues in the alpha-helix. Notably, the propensity of residues in the alpha-helix structure of thermophilic proteins to be soluble is relatively high [15]. It is often assumed on the basis of somewhat anecdotal evidence that thermostable proteins are more readily crystallizable [20]. Remarkably, the property FASG760102 described as “melting point” is at rank 3. All the values of melting point, molecular weight and conformational entropy for all amino acids are listed in Table 6.

**Table 7:** The five top-ranked physiochemical properties in the AAindex database having the highest absolute correlation with crytalizability scores of amino acids.

Rank	AAIndex	Correlation R	Description
1	AURR980101	0.61	Normalized positional residue frequency at helix termini N4′ (Aurora-Rose, 1998)
2	MAXF760106	−0.57	Normalized frequency of alpha region (Maxfield-Scheraga, 1976)
3	FASG760102	0.54	Melting point (Fasman, 1976)
4	NAKH900113	0.54	Ratio of average and computed composition (Nakashima et al., 1990)
5	SNEP660104	−0.53	Principal component IV (Sneath, 1966)

The Pearson's correlation between melting point and crystallizability scores of amino acids is R = 0.54. The correlations between the sequence scores for melting point and crystallizability scores are R_1_ = 0.61 and R_2_ = 0.90. The amino acids with high melting points are crystalline solids. Consider Glu and Ala with high propensity scores of crystallizability and solubility mentioned above. The melting point 249°C of Glu is relatively low ranked at 13, compared with that of Ala (297°C) ranked at 3. Ala with a high melting point and a small molecular weight (at rank 2) is unusual for small organic molecules. The side chain conformational entropy of individual amino acids reported in [21] is also utilized to give a suggestion of considering the mutation point for enhancing crystallizability [22].

Smaller amino acids are reasonably selected as candidates in enhancing crystallizability because they have lower conformational entropy to mediate crystal contact [14], [23]. It is desirable to know whether the role of small molecular weight is important for enhancing crystallization. The correlations between the molecular weight and crystallizability scores for amino acids and sequences are R =  0.05, R_1_ = −0.12 and R_2_ = 0.30. The results reveal that the molecular weight cannot be the individual factor only considered in the mutagenesis analysis. The correlations between the conformational entropy and crystallizability scores are R = −0.32, R_1_ = −0.4 and R_2_ = −0.6. The inverse correlation R_2_ = −0.6 reveals that the conformational entropy is obviously relative to crystallizability only for the extreme case of crystallizable and non-crystaliizable proteins.

It is hypothesized that mutagenesis of surface residues such as Lys and Glu to Ala or other smaller amino acids might systematically improve protein crystallization, indicated in the study [24]. Large flexible amino acids on the surface, such as Lys, Glu and Gln, constitute an impediment to inter-molecular interaction and consequently to protein crystallization [20]. Glu has high conformational entropy while Ala has the lowest conformational entropy. Considering the propensity scores of crystallizability and solubility, as well as melting point, molecular weight and conformational entropy, it is feasibly hypothesized that the mutagenesis of surface residue Ala has large probability of enhancing crystallizability in a generalized condition for applying protein engineering approaches.

Cys, Asn and Ser have the lowest propensity scores to be crystallizable mentioned above. Ser has small molecular weight, high conformational entropy and a low melting point. Cys has the lowest melting point (rank 20), moderate molecular weight and low conformational entropy. Compared with Ser, Asn has a slightly higher melting point and larger propensity scores of crystallizability and solubility. The mutation of Cys to small molecule can improve protein solubility helping in crystallization [25]. Considering the five factors as those in analysing Ala shown in Table 6, it is hypothesized that Cys has small crystallizability and Ser is slightly better than Cys considering the lowest melting point 178°C of Cys.

### Surface mutagenesis of using Ala, Cys and Ser

Several approaches have been developed to enhance protein crystallizability. With the protein engineering approach to increasing the success rate in crystallization, the substitution of single-site amino acids can dramatically affect the crystallization of proteins. However, it is reported that the question of which substituting residue would perform better than others is more difficult to answer [23]. Many studies further presented advantages of single-site mutations for increasing the solubility of proteins and obtained higher quality of crystals [20].

From the analysis of Table 6, the mutagenesis of surface residue Ala has large probability of enhancing crystallizability as a substituted mutant. The most frequently used mutation of X→Ala (replacing amino acid X by Ala) are Glu→Ala and Lys→Ala from the literature survey. It is reasonable that the mutation of Ala→X in enhancing crystallizability in literature is rare and ineffective. We found one mutation of Ala→Cys which is not effective in enhancing crystallizability [26]. This result of Ala→Cys can be well recognized from the analysis of Table 6. The conformational entropy reduction of surface residues in the surface entropy reduction strategy is considered as a main reason for the X→Ala mutation [23], [24], [27]–[34] where Ala has the lowest conformational entropy. The amino acids Glu and Lys having the (conformational entropy, rank) equal to (1.81, 17) and (1.94, 18), respectively, are frequently replaced by Ala. However, the mutation Lys→Ala in these studies [23], [24], [28]–[33], [35] has larger probability than the mutation Glu→Ala in these studies [23], [27]–[30], [32]–[36] of successfully enhancing crystallizability. This statistic finding can be explained by analyzing the results of SCM that Glu has the largest crystallizability score and the second largest solubility score. In principle, the crystallization of proteins is based on rational mutagenesis of surface residues to create patches with low overall conformational entropy in order to facilitate the formation of crystal contacts [20]. Improving solubility of proteins is another reason for the mutation of X→Ala [37] because of this procedure is necessary in protein crystallization [20] and Ala has the largest solubility score.

We would examine the mutations Cys→X and X→Cys from literature survey where Cys has the ranks of crytallizabilitty, solubility and melting point equal to 18, 17 and 20, respectively. Hence, Cys is possibly the important obstacle for protein crystallization. Most mutations of Cys→X enhanced crystallizability according to the reasons of enhancing protein solubility and decreasing aggregation and molecular size [37]–[42]. Ser is a well-known substituted mutant of Cys because Ser can conserve a similar protein function of Cys [37], [39]–[42]. The mutation Cys→Ala could be the perfect mutation of enhancing crystalizability according to our hypothesis in this study, which is reported as successful enhancement in the study [38]. The mutations of X→Cys are believed to enhance crystallizability for obtaining useful heavy-atom derivation [26], [43]–[45]. However, all mutations of X→Cys in these studies [26], [43]–[45] could not successfully improve crystallizability. This scenario is reasonable according to our hypothesis.

Ser has the lowest crystallizability and solubility scores. Therefore, the mutations of Ser→X should increase the probability of enhancing protein crystallization for the same reason with Cys. We found the mutations of Ser→Cys for obtaining useful heavy-atom derivatives to enhance crystallizability [43]–[45]. However, all these mutations of Ser→Cys in the studies [43]–[45] fail to increase the crystallizability. It might be reasonable that the mutation Cys→X demonstrated the high probability of enhancing protein crystallization [37]–[42]. Relatively few mutations of X→Ser were conducted to enhance crystallizability. However, some results of the mutations X→Ser demonstrated the successful enhancement of protein crystallization [23]. From the previous discussion, the mutations of Cys→Ser resulted in enhancing crystalizability [37], [39], [41], [42]. Furthermore, the replacements of Glu, Lys and Gln are mostly successful for the reason of reducing conformational entropy [23]. The ranks of conformational entropy for Ser, Glu, Lys and Gln are 16, 17, 18 and 20, respectively. Interestingly, from Table 6, Ser is the only one having high conformational energy among the top-five amino acids with the lowest molecular weight (rank 3). The results reported by Cooper et al. [23] showed that both Ser and His residues performed less well, but these two amino acids were better than the wild type in promoting protein crystallization. Further studies are needed to evaluate effectiveness of the mutation X→Ser in increasing the probability of enhancing protein crystallization for some specific proteins and conditions.

### Discussion of rational mutagenesis of surface residues

The problem of which substituting surface residue would perform better than others involves many interior and exterior factors which determine the ability of protein crystallization. Considering different design aims for specific proteins, it is desirous but difficult to accurately determine mutations for enhancing crystallizability. This study investigates mutagenesis analysis based on the estimated scores of crystallizability and solubility using SCM and the biophysical properties of amino acids such as melting point, molecular weight and conformational entropy. The literature survey and our analysis reveal the hypothesis that the mutagenesis of surface residues Ala and Cys has large and small probabilities of enhancing crystallizability served as substituted mutants in a generalized condition. The ranks in terms of propensity scores of amino acids to be crystallizable and related physicochemical properties (Table 6) provide guide information of mutagenesis.

**Figure 4:**
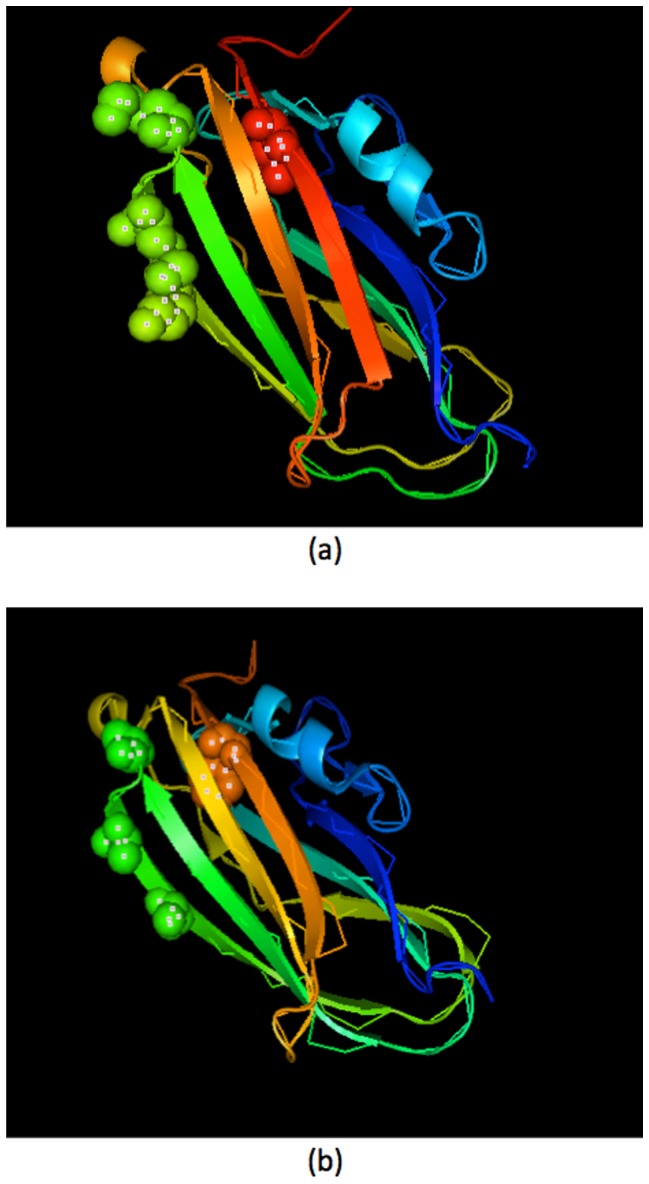
The three-dimensional structure of Rho GDP-dissociation inhibitor. (a) The predicted structure of a wild type Rho GDP-dissociation inhibitor and (b) The structure of a mutant Rho GDP-dissociation inhibitor (NDelta66: K135,138,141A;L196F mutant; 1fso).

Longenecker et al. [24] reported that the Lys→Ala mutations enhanced the crystallization of human RhoGDI mutants compared to the wild type (not crystallizable). Figure 4 shows the three-dimensional structure of Rho GDP-dissociation inhibitor with a) the predicted structure of its wild type obtained by (PS)^2^ (Protein Structure Prediction Server) [46], and b) its NDelta66: K135,138,141A;L196F mutant, 1fso [24], which are generated using PyMOL [47]. Mutation of large flexible surface amino acids to the smaller residues with no conformational entropy might lead to enhancement of crystallization. Additionally, the ranks of Ala are higher than those of Lys in all aspects of propensity scores and biophysical properties. All the results of single and triple mutants support our hypothesis to enhance proteins' ability to crystallize [24].

### Conclusions

We have proposed an ensemble method SCMCRYS for prediction of protein crystallization based on a scoring card method (SCM) with the sequence features of *p*-collocated amino acid pairs. The SCM classifier determines the crystallization of a sequence based on a weighted-sum score. The weights are the composition of the *p*-collocated amino acid pairs, and the propensity scores of the amino acid pairs are estimated using a statistic with optimization approach. SCMCRYS predicts the crystallization using a simple voting method from a number of SCM classifiers. Not like existing prediction methods in pursuit of high accuracy, the SCM-based prediction method aims to maximize both the simplicity and interpretability of used features and classification method. The experimental results show that the SCM-based methods are comparable to the SVM-based methods in terms of accuracy for single and ensemble classifiers.

In this study, we propose the prediction method (SVM_DPC) of using SVM and the dipeptide composition feature, which has the highest accuracy, compared with existing SVM-based single-classifier methods. The result shows that the feature of dipeptide composition play an important role in the estimation of crystallizability. The proposed SCM-based method makes the best use of dipeptide composition in achieving high prediction accuracy and quantifying the dipeptide's crystallizability using the estimated propensity scores of dipeptides to be crystallizable. Although the protein crystallizability is influenced by various condition factors and not easy to estimate, the weighted-sum score of a sequence for determining whether it is crystallizable or not can be served as an index of crystallizability.

Many applications of protein engineering have shown that some effective single-site mutations could dramatically enhance the crystallizability of proteins. However, how to determine the substitution of amino acids for single and multiple mutants is not clear. The crystallizability scores of amino acids for general proteins in a generalized condition are helpful to mutagenesis analysis. Based on the estimated scores of crystallizability and solubility using SCM, melting point, molecular weight and conformational entropy of amino acids, the mutagenesis analysis reveals the hypothesis that the mutagenesis of surface residues Ala and Cys has large and small probabilities of enhancing crystallizability. The SCM-based method has potential ability to generate various propensity scores of dipeptides for predicting protein functions that the features of dipeptide composition play an important role in the prediction.

## Materials and Methods

### Dataset

We obtained the training and test sets containing 3587 and 3585 protein sequences, respectively, from the work [13]. The protein similarity among sequences has been reduced 25% [13]. Two sequences with lengths of 9 and 11 were removed for using the *p*-collocated AA pair (*p* = 0 to 9). We also removed several protein sequences containing special characters, such as X and U. In our experiment, we considered the training set CRYS-TRN and independent test set CRYS-TEST, as summarized in Table 8. CRYS-TRN consists of 1197 crystallizable and 2378 non-crystallizable proteins.

**Table 8:** The datasets for evaluating the predictors of protein crystallization, obtained from Mizianty and Kurgan [13].

Dataset	Number in [13]	Number in this study	Final dataset		
			Positive	Negative	
CRYS-TRN	3587	3575	1197	2378	
CRYS-TEST	3585	3572	1198	2374	

Some sequences of short length and with non-amino acids are removed.

### Scoring card method

The scoring card method (SCM) is a general-purpose prediction method for protein functions from primary protein sequences, especially for the functions that the dipeptide composition plays an important role in determining the functions. The SCM method consists of 1) both positive and negative datasets as input, 2) the statistic method for generating an initial scoring card based on dipeptide composition, 3) derivation of propensity scores of amino acids, 4) the optimization method for refining the scoring card, and 5) establishment of a binary SCM classifier with a threshold value.

The procedure of the SCM method is briefly described below. More details about SCM can be found in [15].

- Step 1. Prepare a training dataset CRYS-TRN consisting of two subsets for positive (crystallizable) and negative (non-crystallizable) classes.
- Step 2. Generate an initial scoring card consisting of 400 propensity scores of dipeptides by using a statistical approach.
  1. Calculate the numbers of 400 dipeptides in each class.
  2. Normalize the dipeptide composition by dividing the numbers using the total numbers of dipeptides in each class.
  3. Obtain the propensity score of each individual dipeptide by subtracting the score of the non-crystallizable sequence from that of the crystallizable sequence.
  4. Normalize the scores of all dipeptides into the range [0, 1000].
- Step 3. Derive the propensity score of each amino acid B by averaging the 40 scores of dipeptides BX and XB where B and X can be any amino acid.
- Step 4. Optimize the dipeptide scoring card (Scard) consisting of 400 scores by using an intelligent genetic algorithm (IGA) [16]. In the chromosome representation, the 400 real-valued variables are encoded in a chromosome of IGA, which is in the range [0, 1000]. The fitness function of IGA is to maximize the prediction accuracy in terms the area under the ROC curve (AUC) [48] and maximize the Pearson's correlation coefficient (the R value) between the initial and optimized scores of amino acids, described as follows (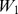 = 0.9 and 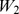 = 0.1 in this study). To avoid from overfitting, a 10-CV assessment is utilized in evaluating the fitness function [15].
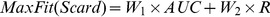
(1)
- Step 5. The prediction of a sequence P bases on the scoring function 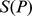, i.e., a weighted-sum score, and a threshold value determined by maximizing the prediction accuracy of using the training dataset.
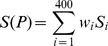
(2)where 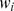 is the frequency of the dipeptide composition of P, and 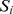 is the score of the i-th dipeptide. P is classified as the positive class when S(P) is greater than the threshold value; otherwise, P is the negative class.

### Ensemble scoring card method

**Figure 5:**
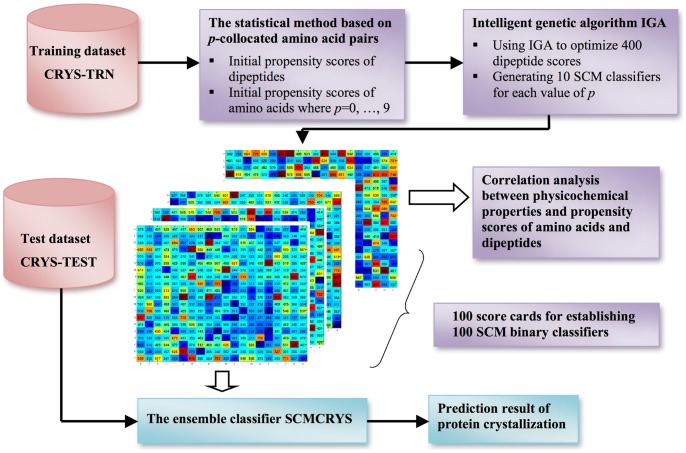
The system flowchart of the SCMCRYS method.

The use of ensembles is a well-known approach to advancing performance in the aspects of prediction accuracy and robustness, especially when the size of the training dataset is not large enough. The proposed ensemble SCM method SCMCRYS utilizes the *p*-collocated amino acid pairs [8] (the collocated dipeptides, as defined in [11]) to predict protein crystallization. Previously, the *p*-collocated AA pairs have been proposed as crucial features for improving predictive performance [8], [11]. For CRYSTALP2, the largest value of *p* used is four. In this study, *p* = 0, 1, …, 9. The SCM method of utilizing the *p*-collocated AA pairs (*p*≠0) is similar to the SCM method using dipeptides (*p* = 0). The system flowchart of SCMCRYS is shown in Figure 5.

Due to the use of randomicity mechanism, genetic algorithms are characterized as a non-deterministic method that the results of all independent runs are not the same. Therefore, 10 independent runs were conducted to generate 10 SCM classifiers from optimizing the scores of the Init-SCM method for each value of *p* where *p* = 0, 1, …, 9. The SCMCRYS method predicts an unknown protein by taking a majority vote of 100 SCM classifiers.
